# Headache in Patients with Cervical Spondylotic Myelopathy

**DOI:** 10.1155/2020/8856088

**Published:** 2020-09-28

**Authors:** Shoji Yabuki, Kozue Takatsuki, Koji Otani, Takuya Nikaido, Kazuyuki Watanabe, Kinshi Kato, Hiroshi Kobayashi, Jun-ichi Handa, Shinichi Konno

**Affiliations:** ^1^Department of Orthopaedic Surgery, Fukushima Medical University School of Medicine, 1 Hikarigaoka, Fukushima 960-1295, Japan; ^2^Department of Pain Medicine, Fukushima Medical University School of Medicine, 1 Hikarigaoka, Fukushima 960-1295, Japan

## Abstract

**Purpose:**

The anatomical mechanisms of cervicogenic headache caused by upper cervical lesions have been reported. However, the pathomechanisms of headache caused by lower cervical spine disorders remain unknown. The purpose of the current study was to clarify the prevalence and pathogenesis of headaches in patients with cervical spondylotic myelopathy (CSM).

**Methods:**

In this retrospective study, a questionnaire regarding preoperative and postoperative symptoms was sent to 147 patients with CSM who were surgically treated in our hospital during the previous 10 years. All of the surgical procedures were decompression surgeries between the C3 and C7 levels. Data from 74 patients (50.3%) were available for analysis. Subjects were divided into four groups according to the presence or absence of preoperative and postoperative headache. The severity of pain, severity of neuropathic pain symptoms, depression, severity of myelopathy, and quality of life (QOL) were also evaluated using questionnaires. The scores of these questionnaires were then compared between the four groups. Kruskal–Wallis tests with Dunn–Bonferroni *post hoc* tests were used for comparisons.

**Results:**

Of the patients with CSM, 31% had headaches preoperatively, and 43% of these headaches disappeared postoperatively. Type 4 (preoperative headache-positive/postoperative headache-positive) patients had more severe pain and neuropathic pain symptoms and lower QOL scores compared with type 1 (preoperative headache-negative/postoperative headache-negative) patients.

**Conclusions:**

Approximately one-third of all patients with CSM had headaches preoperatively. Headache in patients with CSM may be neuropathic pain. A proportion of headaches in patients with CSM can be treated by decompression surgery.

## 1. Introduction

The concept of cervicogenic headache was introduced by Sjaasatd in 1983 [1]. He suggested diagnostic criteria and revised them in 1998 [2]. In 2004, the International Headache Society also published diagnostic criteria [3]. Bogduk and Govind [4] first reported the anatomical mechanisms of headache caused by upper cervical lesions. There is no direct neuroanatomical link between the lower cervical afferents and the trigeminocervical nucleus. These authors therefore concluded that intermediate mechanisms, such as muscle tension and secondary kinematic abnormalities that affect upper cervical joints, might be involved in headache caused by lower cervical disorders [4]. Several studies have reported the clinical observation that headache can be relieved by surgical treatment for radiculopathy, myelopathy, or neck pain caused by lower cervical lesions [5–7]. Furthermore, Persson et al. [8] evaluated headaches in patients with cervical radiculopathy using selective nerve root blocking. Their results suggest that cervical root compression from degenerative diseases in the lower cervical spine, producing radiculopathy, might also induce headaches [8]. However, the pathomechanisms of headache caused by lower cervical spine disorders remain unknown.

The purpose of the current study was to clarify the prevalence and pathogenesis of headache in patients with cervical spondylotic myelopathy (CSM). CSM is a disease with various symptoms, such as numbness of the upper and lower extremities, disturbed fine motor skills, and gait disturbance. These symptoms are caused by spinal cord compression as a result of degenerative changes in the intervertebral discs, vertebrae, and ligamentum flavum.

## 2. Materials and Methods

**Figure 1:**
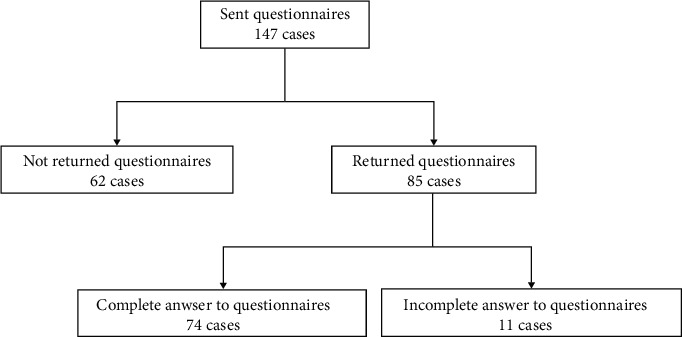
Flow chart of subjects.

This retrospective study was approved by the ethical committee of Fukushima Medical University (no. 1015). We sent questionnaires regarding preoperative and postoperative symptoms to 147 patients with CSM who had been surgically treated in our hospital during the previous 10 years (2001–2010). This survey was performed from September 2011 to January 2012. All of the surgical procedures were decompression surgeries between the C3 and C7 levels (segmental laminotomies or laminoplasties) [9]. Data from 74 cases (50.3%) were available for analysis (Figure 1). The patients were divided into four groups according to the presence or absence of headache preoperatively (+ or −) and postoperatively (+ or −). Presence or absence of headache was confirmed by questionnaire, regardless of location, frequency, and duration. Headache was not evaluated by Sjaastad's criteria. If the patients had headache preoperatively, we asked postoperative changes of headache. When headache completely disappeared postoperatively, it showed −. On the other hand, when headache still existed or improved but partly remained postoperatively, it showed + (Table 1).

The patients with CSM had numbness and/or pain in the upper and lower extremities. They also complained of disturbed fine motor skills and gait disturbance. The severity of pain in upper and lower extremities, severity of neuropathic pain symptoms in upper and lower extremities, depression, severity of myelopathy, and quality of life (QOL) were evaluated between the groups using the following questionnaires.

### 2.1. Severity of Pain

A short form of the McGill Pain Questionnaire (SF-MPQ) was used for the evaluation of pain. The main component of the SF-MPQ consists of 15 descriptors (11 sensory and 4 affective) which are rated on an intensity scale as 0 = none, 1 = mild, 2 = moderate, or 3 = severe. Three pain scores are derived from the sum of the intensity rank values of the words chosen for sensory, affective, and total descriptors. The questionnaire's interpretation is relatively basic: a higher score means a higher pain level [10, 11]. The SF-MPQ was used with permission.

### 2.2. Severity of Neuropathic Pain Symptoms

Neuropathic pain symptoms were evaluated using the Neuropathic Pain Symptom Inventory (NPSI). The NPSI is a self-administered questionnaire that is specifically designed to evaluate the different symptoms of neuropathic pain. Each of the items was quantified on a numerical scale (0–10). The NPSI includes 10 descriptors (plus two temporal items) that allow the discrimination and quantification of five distinct clinically relevant dimensions of neuropathic pain syndromes [12, 13]. The NPSI can be used without a license.

### 2.3. Depression

The Beck Depression Index (BDI) was used to evaluate depression. This questionnaire measures the self-reported presence and severity of depression symptoms. A higher score indicates greater depression, and the scores can be classified as follows: minimal range = 0–9, mild depression = 10–16, moderate depression = 17–29, and severe depression = 30–63 [14, 15]. The BDI was used without a license. The questionnaire is available to purchase from the following companies in Japan: http://www.saccess55.co.jp/, http://www.chibatc.co.jp, and http://www.nichibun.co.jp/.

### 2.4. Severity of Myelopathy

The Japanese Orthopaedic Association Cervical Myelopathy Evaluating Questionnaire (JOACMEQ) was used for this evaluation. The JOACMEQ is a self-reported questionnaire that is completed by the patient. The major characteristic of this questionnaire is that the evaluation is performed based on patient-oriented outcomes. The JOACMEQ is not a single scoring system, but it consists of the evaluation of five different scores: cervical spine function, upper extremity function, lower extremity function, bladder function, and QOL [16, 17]. The JOACMEQ can be downloaded from the Japanese Society for Spine Surgery and Related Research (https://ssl.jssr.gr.jp/member/) and used without a license.

### 2.5. QOL

QOL was evaluated using the MOS Short-Form 36-Item Health Survey (SF-36). This questionnaire is a multi-item, generic, health-related QOL survey intended to measure “general health concepts not specific to any age, disease, or treatment group.” The SF-36 measures eight health domains: physical function (PF), role physical (RP), bodily pain (BP), general health (GH), vitality (VT), social functioning (SF), role emotional (RE), and mental health (MH). The SF-36 can also be used to evaluate the physical component summary (PCS) and mental component summary (MCS) in the summary scores. The scale scores are calculated by summing responses across the scale items, and these raw scores are then transformed to a 0–100 scale. Computerized scoring algorithms are available and can produce norm-based *T* scores for each scale (with a mean of 50 and standard deviation (SD) of 10) [18]. The SF-36 was used under license between our institute and iHope QOL (https://www.sf-36.jp/index.html).

### 2.6. Statistical Analysis

A normality test of the data was performed using the Shapiro–Wilk normality test. Differences in age between the three patient types were analyzed using one-way analysis of variance (ANOVA). Gender bias was analyzed using Pearson's chi-squared test. The questionnaire scores were compared between the three patient types using the Kruskal–Wallis test with the Dunn–Bonferroni *post hoc* test. A *p* value less than 0.05 was considered statistically significant. All analyses were performed using IBM SPSS statistical software (version 26.0, SPSS Inc., Chicago, IL, USA).

## 3. Results

The patients were divided into four types (Table 1). In total, 31% (23/74 cases) of patients with CSM had headaches preoperatively, and 43% (10/23 cases) of these headaches disappeared postoperatively. Only three patients were classified as type 2 patients. We therefore analyzed types 1, 3, and 4 patients only. There were no significant differences in age between the three patient types (*p*=0.208; mean age in years (SD): type 1: 72.0 (12.6), type 3: 65.1 (6.9), and type 4: 68.5 (12.3)). However, there were significant differences in sex between the three types (*p* < 0.05) (Table 1).

### 3.1. Severity of Pain

**Figure 2:**
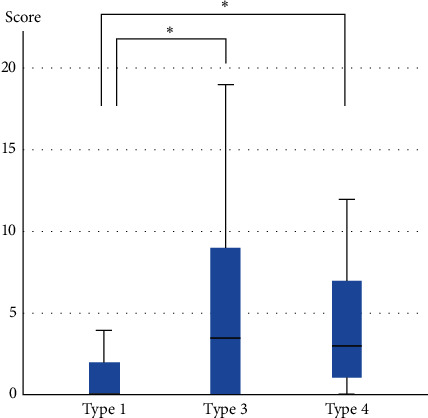
McGill Pain Questionnaire scores in each patient type. The scores of type 3 and type 4 patients were significantly higher than those of type 1 patients. ^*∗*^*p* < 0.05.

The MPQ scores were significantly higher in type 3 and type 4 patients than in type 1 patients (Table 2 and Figure 2).

### 3.2. Severity of Neuropathic Pain Symptoms

**Figure 3:**
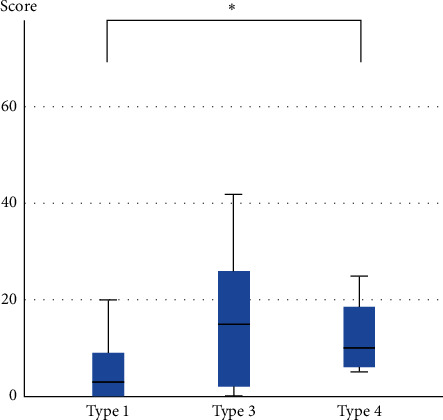
Neuropathic Pain Symptom Inventory scores in each patient type. The scores of type 4 patients were significantly higher than those of type 1 patients. ^*∗*^*p* < 0.05.

The NPSI scores in type 4 patients were significantly higher than in type 1 patients (Table 2 and Figure 3).

### 3.3. Depression

There were no significant differences in BDI scores between the three patient types (Table 2).

### 3.4. Severity of Myelopathy

In the JOACMEQ, there were no significant differences in cervical spine function, upper extremity function, lower extremity function, bladder function, or QOL between the three patient types (Table 2).

### 3.5. QOL

**Figure 4:**
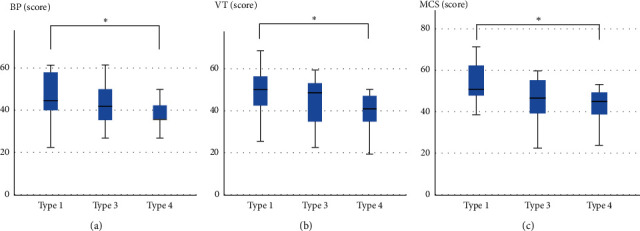
MOS Short-Form 36-Item Health Survey scores in each patient type. The BP (a), VT (b), and MCS (c) scores in type 4 patients were significantly lower compared with those of type 1 patients. ^*∗*^*p* < 0.05, BP: bodily pain, VT: vitality, MCS: mental component summary.

The BP, VT, and MCS scores in type 4 patients were significantly lower than those in type 1 patients (Table 2, Figure 4).

## 4. Discussion

The CSM patients with preoperative headache had higher pain levels compared with the CSM patients without preoperative and postoperative headache, regardless of whether they also had postoperative headache. In addition, the CSM patients with preoperative and postoperative headache had higher neuropathic pain symptom and lower QOL scores compared with the CSM patients without preoperative and postoperative headache.

CSM is a disease with various symptoms, such as numbness of the upper and lower extremities, disturbed fine motor skills, and gait disturbance, caused by spinal cord compression. It has been reported that approximately 40% of patients with cervical myelopathy have severe allodynia or hyperalgesia in the upper and lower extremities [19, 20]. Although the mechanisms underlying these symptoms remain unknown, they are thought to be similar to the mechanisms of spinal cord injury (SCI) pain because repeated minor trauma may be related to cervical myelopathy. That is, allodynia or hyperalgesia in the upper and lower extremities of patients with CSM is most likely neuropathic pain caused by SCI.

The International Spinal Cord Injury Classification mentions pain subtypes in neuropathic pain: at-level SCI pain and below-level SCI pain. Headache is classified as nociceptive pain [21, 22]. However, the results of the current study suggest that headache in patients with CSM might be related to neuropathic pain. If the trigeminocervical nucleus is a central nucleus of headache caused by cervical spinal cord injury, then central sensitization in the injured spinal cord may relate to this mechanism. Sufficient levels of ectopic firing at the injured spinal cord may transmit to the trigeminocervical nucleus because the cervical spinal cord is close to the trigeminocervical nucleus. However, these mechanisms need to be clarified by experimental and clinical studies in the future.

In the present study, 31% of patients with CSM had headache preoperatively, and these patients had more severe neuropathic pain symptoms than the other patients. Furthermore, 43% of these patients reported that headache disappeared after decompression surgery. These findings suggest that headache in patients with CSM may be associated with spinal cord compression. That is, headache in patients with CSM may be a kind of neuropathic pain caused by spinal cord compression. It is therefore important to pay attention to headache in patients with CSM and to clarify which patients can be treated surgically for headache.

There are several limitations to the current study. First, this study was retrospective. The preoperative characteristics of headache and the course of postoperative changes of headache were therefore unable to be clarified. Second, the rate of analyzed patients was low (50.3%). The prevalence of headache and the improvement rate of headache after surgery in our study might therefore differ from the total patient population. Finally, we were unable to investigate the diagnostic procedures for evaluating headache originating from facet joints or soft tissue. However, the current study did present the possibility that the mechanism of headache in patients with CSM might be neuropathic pain and suggest that some headache in patients with CSM can be treated surgically.

## 5. Conclusions

Headache existed in 31% of the patients with CSM in the present study, and these headaches might be associated with a neuropathic pain mechanism. Furthermore, 43% of patients with CSM reported that headache disappeared following decompression surgery.

## Figures and Tables

**Table 1:** Classification of the subjects according to whether there is headache preoperatively and postoperatively.

Type	Preoperative headache	Postoperative headache	N (male/female) (cases)
1	−	−	48 (36/12)
2	−	+	3 (3/0)
3	+	−	10 (4/6)
4	+	+	13 (11/2)

−: absence, +: presence. When headache completely disappeared postoperatively, it showed − (type 3). On the other hand, when headache still existed or improved but partly remained postoperatively, it showed + (type 4).

**Table 2:** Results of various questionnaires in each type.

		1 (*n* = 48)	3 (*n* = 13)	4 (*n* = 10)	*p* value
MPQ		0 (0–2)	3.5 (0–9.8)	3 (0.5–7.5)	0.01
NPSI		3 (0–9)	15 (1.5–28.8)	10 (6–25)	0.02
BDI		5.5 (1–14.8)	11 (5.8–24.8)	13 (10.5–20)	0.51
JOACMEQ	Cervical spine function	75 (43.8–100)	60 (40–100)	35 (10–85)	0.15
	Upper extremity function	86.5 (56.8–100)	95 (63–95)	79 (53–89)	0.57
	Lower extremity function	66 (55–100)	68 (55–86)	64 (18–86)	0.87
	Bladder function	75 (50–94)	75 (44–88)	50 (19–81)	0.23
	QOL	57 (39–69.3)	57 (29–70	47 (18–50)	0.06
SF-36	PF	34 (22.5–51.6)	37.1 (28.7–45.5)	37.5 (27–44.6)	0.97
	RP	35.8 (25.6–49.4)	42.6 (22.2–46)	35.8 (25.6–46)	0.99
	BP	44.6 (39.7–61.4)	42 (34.2–49.9)	35.6 (35.3–43.5)	0.04
	GH	43 (37–52.3)	37 (30.9–44.9)	39.7 (28.2–43.2)	0.09
	VT	50.2 (41.8–56.4)	48.7 (34.9–53.3)	41 (30.3–48.7)	0.04
	SF	50.5 (37.4–57.1)	40.7 (29.2–52.2)	43.9 (34.1–53.8)	0.33
	RE	39.6 (31.1–56.6)	39.6 (24.7–50.2)	39.6 (26.9–43.8)	0.59
	MH	51.8 (41.1–57.1)	49.1 (31.2–57.1)	43.8 (35.9–49.1)	0.10
	PCS	(20.9–48.2)	39.2 (26–43.5)	31.8 (10.9–40.7)	0.65
	MCS	51 (1–14.8)	46.7 (36.7–56.8)	45 (37.2–52.6)	0.01

Data were shown as median (interquartile range (IQR)). MPQ: McGill Pain Questionnaire, NPSI: Neuropathic Pain Symptom Inventory, BDI: Beck Depression Index, JOACMEQ: Japanese Orthopaedic Association Cervical Myelopathy Evaluation Questionnaire, QOL: quality of life, SF-36: MOS Short-Form 36-Item Health Survey, PF: physical functioning, RP: role physical, BP: bodily pain, GH: general health, VT: vitality, SF: social functioning, RE: role emotional, MH: mental health, PCS: physical component summary, and MCS: mental component summary.

## Data Availability

The datasets used and/or analyzed during the current study are available from the corresponding author on reasonable request.
